# A qualitative interview study of patient experiences of receiving motivational enhancement therapy in a Swedish addiction specialist treatment setting

**DOI:** 10.1186/s13722-023-00398-7

**Published:** 2023-07-20

**Authors:** Stina Ingesson Hammarberg, Jennie Sundbye, Rebecca Tingvall, Anders Hammarberg, Christina Nehlin

**Affiliations:** 1 Centre for Psychiatry Research, Department of Clinical Neuroscience, Karolinska Institutet, & Stockholm Health Care Services, Norra Stationsgatan 69, 113 64 Region Stockholm, Sweden; 2 Division of Psychiatry, Uppsala University Hospital, Uppsala, Sweden; 3 Department of Medical Sciences, Psychiatry, Uppsala University, Uppsala, Sweden

**Keywords:** Motivational enhancement therapy, Alcohol use disorder, Qualitative, Thematic analysis

## Abstract

**Background:**

Motivational enhancement therapy (MET) has shown to be efficacious as treatment of alcohol use disorder (AUD), in reducing alcohol consumption and related consequences. However, qualitative research on how patients perceive this treatment is lacking. The aim of this study was to explore how patients experience MET as a treatment for AUD.

**Methods:**

Fifteen patients (8/7 female/male) participated in semi-structured interviews after receiving MET at a specialized addiction outpatient clinic in Sweden. Data were analyzed by thematic analysis.

**Results:**

Five themes were identified: the therapist conveyed the MI-spirit, the therapist did not guide on how to reach the goal, participants were committed to change before starting treatment, participants were uncertain if treatment was enough to maintain change, and significant others were not wanted in sessions. Participants appreciated the supportive relationship with their therapist, but some experienced therapy as overly positive, with no room to talk about failure. Further, they experienced a low level of guidance in goal-setting. For some, this was empowering, while others requested more direction and advice. Participants perceived their motivational process to have started before treatment. MET was considered to be too brief. None of the participants brought a significant other to a session.

**Conclusions:**

Therapist behaviors in line with MI spirit were emphasized as key to the development of a positive therapeutic relationship. More specific advice on goal-setting may be effective for supporting change in some patients. Longer treatment is requested among patients to support the patient’s self-efficacy for change. Significant others can support change without necessarily being present in sessions.

*Trial registration*: The current trial was retrospectively registered at isrtcn.com (14539251).

## Introduction

Motivation is essential for a person to achieve behavior change and maintain goal-related behaviors [1]. To increase motivation and thus support people to change problematic alcohol use, the American psychologist William Miller introduced motivational interviewing (MI) in 1983 [2]. Rather than being a distinct form of therapy with fixed interventions, MI aims to explore and resolve ambivalence that people might have about changing problem behaviors. Since then, the approach has been further developed as a conversation style for strengthening a person’s own motivation, commitment, and self-efficacy to change [1]. An important concept in MI is that of a particular MI spirit, which includes encouraging collaboration, conveying acceptance, showing empathy, and evoking the patient’s own reasons for change [1].MI is mainly used in healthcare and social service settings, in brief interventions addressing, e.g., alcohol or drug use, gambling or smoking cessation, but also in supporting individuals to engage in treatment that promotes health [3, 4].

Since the introduction of MI more than 30 years ago, several meta-analyses have been conducted to investigate its effectiveness [4–7]. Treatment effects are small to medium-sized compared to untreated controls, but effect sizes vary substantially between studies. These variations have called for a better understanding of which MI features are associated with client behavior change. This has resulted in extensive quantitative research on therapist skills and what specific therapist behaviors produce change statements from the patient, which in turn is predictive of positive clinical outcomes [8–11].

Motivational enhancement therapy (MET) is a manualized version of MI for the treatment of alcohol use disorders (AUD) that was introduced in the 1990s as one of three interventions investigated in Project MATCH [12]. MET has been shown to be efficacious in reducing alcohol consumption and related consequences in several large-scale randomized controlled studies [12–14]. MET typically includes three or four treatment sessions [12, 13, 15]. At the first session, time is dedicated to structured feedback on an initial assessment, which serves as a basis for further discussion of the patient’s desired change. The following treatment sessions are dedicated to identifying and following up on the patient’s change goals [15, 16]. Patients are encouraged to invite a significant other to take part in the first session in order to support the change process.

Although outcomes of MI and MET have been studied extensively, studies involving patient perspectives on the treatments are lacking [17]. One possible approach to studying patient experiences of MI treatment is to use qualitative research methods. There are only a few studies investigating patient experiences of MI in individuals with AUD. Among these, different methodological approaches have been applied. In one study investigating the views of individuals with problem drinking on receiving a brief intervention based on MI when admitted to an orthopedic ward, potential enablers or barriers to changing alcohol consumption were identified [18]. Patients emphasized the cost–benefit balance of change versus no change and personal gains of the change process, as well as future challenges such as fear of relapse. In another study on adult problem drinkers, patients watched a recording of one of their MI sessions and were then instructed to describe important moments of the session [19]. The patients pointed out the importance of the relationship with the therapist, and therapist behaviors that supported the therapeutic alliance, such as a non-confrontational and non-judgmental approach and the patient being treated as an equal. Further, patients emphasized it as helpful that they could be honest and open about their problem. A secondary analysis from the United Kingdom Alcohol Treatment Trial (UKATT) analyzed patients’ and therapists’ perceptions on what was helpful in MET treatment [20]. Patients and therapists answered in written statements what was least and most useful, after the completion of the sessions. Patients mentioned several positive aspects, e.g., having someone to talk to and that sessions increased awareness and commitment to change. More specifically, the patients who attended MET appreciated when they received feedback from the therapist and also that the treatment focus was on the future. The least helpful aspects were the session structure and the repetitiveness of the sessions.

Taken together, MET is scientifically proven to be effective in terms of reducing alcohol consumption and related consequences. Despite this, patient experiences of MET are not well-investigated, more specifically as regards how the main aim of MET—to support and maintain change—is achieved from the patient perspective. In the current study, the aim was to investigate patient experiences and thoughts on participating in MET. Further, the aim was to explore which treatment features, if any, were perceived as helpful, and if patients experienced that MET treatment was sufficient to achieve the desired change in drinking behavior.

## Methods

### Participants and procedure

Participants were recruited within a randomized controlled trial (RCT) in Stockholm, Sweden, that investigated the efficacy of two psychological treatments: Behavioral Self-control Training (BSCT) and MET, including 250 individuals with AUD and a goal of controlled drinking. The primary outcome of the trial has been reported elsewhere. For detailed information on participants and study procedures in the RCT, see Ingesson Hammarberg, in press. The original trial was approved by the Regional Ethics Board in Stockholm (DNR: 2016/634-31/2). The current study was approved by the Regional Ethics Board, as an amendment to the original study. The trial was retrospectively registered in ISRCTN (14,539,251) (05/06/2018). The study was conducted in accordance with the Declaration of Helsinki and was reported in accordance with the Consolidated criteria for Reporting Qualitative research) (COREQ) checklist [21].

All patients underwent assessment as part of the clinical trial protocol, including of level of alcohol consumption, alcohol-related consequences, and psychiatric diagnostics. Inclusion criteria were: a stated goal of controlled drinking, fulfilment of a diagnosis of alcohol use disorder, and age 18–70 years. Exclusion criteria were: fulfilment of any other substance use disorder except nicotine, severe psychiatric comorbidity, and severe somatic risk related to continuous alcohol consumption.

Participants in this study were recruited during 2021, among those who had received MET and attended the 26-week follow-up in the RCT. If consenting, participants took part in an interview either via a video meeting platform or by telephone. All participants gave verbal and written consent to participate and to data being used in a scientific publication. The interviews were audio-recorded and then transcribed by the interviewers. None of the participants withdrew consent after being interviewed and there was no reimbursement for their participation.

### Motivational enhancement therapy

MET, as applied in this study, included four sessions, and was distributed over 12 weeks [15]. Patient assessment was conducted by an assessor as part of trial routines. Thereafter, at the first treatment session, the assigned therapist offered feedback on this assessment. Due to the COVID-19 pandemic, the majority of participants received their treatment sessions via video meetings. The manual also included two worksheets with the themes (1) change plan and (2) how to maintain change. These could be used within a session or as homework assignments.

### Therapists

Five MET therapists were involved in the current study. All had extensive training in MI and MET, with clinical experience in both MI and MET ranging from 10 to 20 years. Therapists attended regular supervision meetings and received regular feedback on their recordings in accordance with the Motivational Interviewing Treatment Integrity Code (MITI) protocol 4.2.1 [22]. All sessions of which therapists had patients’ consent were recorded to assess treatment integrity in the conducted treatments. A total of 10% of the recorded MET sessions were randomly chosen and scored in line with the Motivational Interviewing Treatment Integrity code (MITI) 4.2.1 by an external expert team. The four areas which were evaluated were; (1) Technical score (average of cultivating change talk + softening sustain talk/2); (2) Relational score (average of empathy + partnership/2); (3) Percentage of complex reflections of all reflections and; (4) Reflection to question ratio) [22].

### Measures

#### Baseline measures

Baseline measures, as well as sociodemographic data, were collected as part of the clinical trial protocol. Alcohol consumption was assessed using the timeline follow-back method [23] over a period of 90 days, in number of standard drinks per day (12 g of pure ethanol) at baseline and follow-up.

#### Interviews

A semi-structured interview guide was developed by the research team. Four areas were covered: (1) The treatment experience, and if there were positive and negative features. (2) Whether the treatment was helpful and, if so, in what way? If not, in what way? (3) Was the treatment sufficient to achieve change regarding alcohol consumption? To what extent? In what way? (4) Thoughts about involving a significant other (SO) in the treatment. The guide was discussed after the first interview and was not changed thereafter.

The interview guide allowed for participants to freely express opinions and permitted follow-up questions from the researcher. The approach to data was inductive, as research questions and interview guide were not chosen based on a specific theory or concept that we wanted to explore. Rather, they were based on our interests and clinical experiences on how patients perceived the therapeutic intervention.

### Researchers’ stance

Interviews were carried out and transcribed by two of the authors (JS and RT). Both are female, trained clinicians (nurse and psychologist), well-acquainted with the practices of MET, but neither was a therapist at the clinic where treatments were conducted. Authors JS and SIH (both female) were both active as research coordinators in the RCT, and acquainted with the individuals who participated in the study. Authors SIH (female) and AH (male) are both trained MI therapists, but were not active as therapists in the current project. The authors’ pre-understanding of MI/MET as clinicians at the treatment site could potentially have had an impact on the analysis. The two-legged position as both therapists and researchers was key in critically evaluating and interpreting data. Lastly, author CN (female) is a senior researcher with long experience of qualitative research. CN is affiliated with a different university and was therefore not involved in the data collection process, or active as a therapist at the clinic, which allowed for a more distanced view of the data.

### Methodological approach

Thematic analysis in accordance with the six phases defined by Braun and Clarke [24, 25] was used in the current study. Transcripts were read independently and repeatedly by the authors, with the research questions in mind. Sentences and meaning units in line with the aims of the study were coded and, after joint discussion, sorted into preliminary themes. The material was re-read and themes were reviewed. Analysis continued until all themes were deemed to be clearly defined and distinct from one another. All authors discussed the coding of data until a consensus was reached and themes were perceived as concisely describing the content. Once 15 interviews had been conducted, information from the final ones did not produce any changes to the themes. Therefore, saturation was deemed to have been achieved [26].

## Results

### Participants

A total of 15 participants who participated in the 26-week follow-up were interviewed. The follow-up rate in the current trial at 26 weeks was 83.6%. Interviews lasted 18–43 min (median 27 min). For more specific details on participants’ characteristics, see Table 1.

**Table 1:** Baseline clinical characteristics and demographic data

Gender (percentage male)	(n = 15)	
	46	
	Mean	SD
Age (years)	55.0	11.4
Alcohol use disorder (DSM-5 criteria fulfilment)	5.3	1.9
AUDIT	20.7	3.9
Mean weekly alcohol consumption^a^	26.0	12.8
Drinks per drinking day^a^	5.8	2.3
Depressive symptoms (MADRS-S)	9.0	6.9
Symptoms of anxiety (GAD-7)	2.8	3.3
Number of sessions attended	4.4	0.9
Educational level	%	
Up to post-secondary education	26.1	
University < 3 years	23.5	
University > 3 years	50.4	
Occupational status		
Employed/Self-employed	83.0	
Retired	11.0	
Unemployed/Sick leave	6.0	
Marital status		
Married/Partner	74.0	
Divorced/Widowed	7.7	
Single	18.3	

*DSM-5* Diagnostic and Statistical Manual of Mental Disorders, 5^th^ edition, *AUDIT* Alcohol Use Disorders Identification Test, *MADRS-S* Montgomery Asberg Depression Rating Scale-Self Rated, *GAD-7* Generalized Anxiety Disorder 7-item. *measured in standard drinks (12 g of ethanol)

### MITI-scoring results

The analysis of MITI scores among the involved therapists resulted in the following mean scores (including cutoffs for the accepted level according to the MITI in square brackets); (1) Technical score = 3.2 (SD = 0.79), [> = 3.0]; (2) Relational score = 3.9 (SD = 0.53), [> = 3.5]; (3) Percentage of complex reflections = 0.6 (SD = 0.14), [> = 0.4]; (4) Reflection to question ratio = 3.3 (SD = 3.3), [> = 1.0]. All results of MITI scoring were above the threshold of acceptable levels of adherence to MITI-protocol [22].

### Analysis

Five themes were identified (example of themes, see Table 2): (a) the therapist conveyed the MI spirit; (b) the therapist did not guide on how to reach the goal; (c) participants were committed to change before starting treatment; (d) participants were uncertain if treatment was enough to maintain change and; (e) significant others were not wanted in sessions. These themes are presented below, with verbatim quotes to illustrate the findings.

**Table 2:** Examples of the analysis process

Quote	Code	Theme
*‘Being able to be completely open relieves the burden of having something that you are covering up a bit. And the shame connected to this, the social shame of having this (alcohol) problem.’*	Thoughts on treatment	The therapist conveyed the MI spirit
*‘To take that step and actively join a treatment, it has a huge impact. Because once you make up your mind, then you’re sort of halfway there, quite a lot of the motivation is already there. And the treatment helps you stay motivated.’*	Sufficient to achieve change	*Participants were committed to change before starting treatment*
*‘She would have taken over and it would have become another type of treatment. I think that wouldn’t have worked out as well for me.’*	Thoughts on treatment	*Significant others were not wanted in sessions*

### Theme: The therapist conveyed the MI spirit

Participants were generally positive when describing their overall experience of the treatment. They emphasized their relationship with the therapist as important to their overall experience of being in treatment. They appreciated that the therapist was supportive of their goals and that he/she created a safe and secure environment that increased their own ability to engage in treatment. A neutral and non-judgmental atmosphere allowed them to verbalize their thoughts and feelings about their drinking problems. Participants also underlined that it was a relief to confide in someone and be able to be completely honest. They appreciated talking to someone professional, who was an expert, and not someone from their personal network.

*“I felt very good during our sessions, and the best thing was that there was no judgment in any way.” (P#15)*

*“Being able to be completely open relieves the burden of having something that you are covering up a bit. And the shame connected to this, the social shame of having this (alcohol problem.” (P#10).*

Although therapists did not express their own opinions, some participants described a high level of emotional support from their therapist. Even if they failed in one ambition (e.g., to reduce the number of drinking days), the therapist focused on and encouraged what had been positive in the specific situation. This helped participants to keep trying instead of giving up or feeling like a failure. However, some participants perceived the encouragement to be a bit exaggerated and appreciated talking more about things that did not work out well.

*“Sometimes it feels like everything is just so very positive, even though it’s not. It’s like, nothing else is ever said.” (P#3).*

*“It was very little that was about something negative really (…) I don’t know, but sometimes you might need that too.” (P#4).*

### Theme: The therapist did not guide on how to reach the goal

It was clear to participants that the therapists wanted them to reflect on their desired goal in treatment themselves, and that the therapists strived to maintain this focus throughout the sessions. Despite this goal orientation, participants felt that they received a low level of guidance on how the goal would be accomplished. To some, this was experienced as if the therapist was not supposed to or allowed to share his/her own opinion of the patient’s situation. Low guidance was perceived as the absence of a specific formula on how changes can be made. To some participants, the low level of guidance created and strengthened a feeling of being the owner of the problem. One participant found the low level of guidance annoying at first, but then perceived it to be part of the method and came to appreciate the therapist’s style.

*“Pretty soon I realized that this will be up to me, that it is up to me to come up with what I’ll do. And that has proven to be good for me. I would have thought that I would get some form of guidelines. At first, I felt a bit lost in this, but then I felt great support, but also, that help is found within yourself.” (P#9).*

*“I had the feeling that I still owned the question and that she (the therapist) was there as a resource to me. (…) When I asked for advice, I got it, and otherwise she mainly encouraged me to think by myself, and to ask myself*: ‘What can I do to improve my situation?’.” (P#13).

Other participants experienced the level of guidance to be insufficient. They found the therapist to be too neutral in their approach. Instead, they would have preferred more specific direction on what goal to pursue and how to pursue it, as well as confirmation from the therapist if the choice of strategy to reach the goal was “right”. Some participants also expressed a wish for more examples and specific advice on how other people managed their problems. Participants who received explicit advice on what strategy to use in a specific situation found this helpful.

*“You need to come up with everything yourself, sort of. What was lacking was some advice I guess; what others have done.” (P#3)*

*“(The therapist) took a very distanced position to what I did, like—this is entirely up to you and nothing like, well done or let’s do this.” (P#12)*

### Theme: Participants were committed to change before starting treatment

Participants repeatedly expressed that their own wish to change was essential for change to occur, and highlighted that they themselves had acted to get help. They described having decided, or even started, to change their drinking behavior before contacting the clinic. The treatment was described as well-suited to participants who felt that they were already in the change process and who were about to initiate specific steps towards that change. Being in treatment was described as consolidating this ongoing change.

*“To take that step and actively join a treatment, it has a huge impact. Because once you make up your mind, then you’re sort of halfway there, quite a lot of the motivation is already there. And the treatment helps you stay motivated.” (P#3).*

### Theme: Participants were uncertain if treatment was enough to maintain change

In general, participants perceived that they needed time to achieve their goals. Though they described the treatment as helpful, they were hesitant to say whether it was sufficient to achieve their desired change. Recurrently, participants reported that the treatment had felt too brief and that they would have wished for additional sessions to maintain their new behavior and/or to manage the risk of lapses of unwanted drinking.

*“I am very disappointed that the treatment does not continue. Because it is like you start something that will change your life and when it feels like you are a bit near the best result, then you are released with no further support. (…) There was no time to establish the routines to manage by oneself again.” (P#2).*

*“I felt like, was that it? I don’t know what it (treatment) was all about, but I thought it was a bit short, for being a treatment.” (P#5)*

### Theme: Significant others were not wanted in sessions

None of the participants reported having brought a SO to any of the treatment sessions. The reasons for not bringing a SO differed—some did not have a person close enough to bring, others were already having a dialogue with a SO. In general, involving a SO was perceived as something positive and as good support, even if the SO had not joined them in treatment.

*“Discussing ideas on strategies with my partner is good support. I believe that it strengthened our relation, as my partner feels that I am taking this seriously and can support me and understand more what I am going through.” (P#10).*

Some participants expressed a fear that bringing their SO would affect both the treatment and their relationship with the SO negatively. Participants also described the treatment as being their own business.

*“She would have taken over and it would have become another type of treatment. I think that wouldn’t have worked out as well for me.” (P#13).*

*“I had a need to make it on my own. And to formulate and find motivation on my own.” (P#8)*

One participant reflected that bringing a SO to treatment could have led to a positive result, but if their attendance was mandatory, the participant would have felt greater resistance to seeking treatment.

## Discussion

The aim of the current study was to investigate how patients who were treated with MET perceived their treatment and if it was sufficient to achieve the desired change. To our knowledge, this is the first qualitative interview study that explores patient experiences of receiving MET for AUD. Participants reported the therapist relationship as important for feeling secure and engaging in treatment. However, a lack of guidance on how to reach personal goals, as well as what would be an appropriate goal, was perceived as less helpful to some. Participants also expressed a wish for a longer treatment period, to increase their self-efficacy to change. Lastly, participants were hesitant or even negative towards bringing a SO to the treatment sessions.

Participants described a positive, supportive, and non-judgmental relationship with the therapist as a particularly important aspect of the treatment. These are specific qualities suggested to represent MI spirit [27]. Conveying the MI spirit may be described as a two-sided skill: both delivering supportive therapist behaviors, such as open-ended questions and complex reflections and affirmations, and suppressing confrontational comments and the impulse to talk instead of listen or to offer advice [10]. It is not known if it was MI-specific therapist skills that supported the development of the positive relationship described in the current study. Still, other MI studies have transferable results on what elements in treatment were perceived as helpful by patients; e.g., having a collaborative, non-judgmental, and supportive relationship [10, 19, 28]. One aspect of the relationship with the therapist was having someone professional outside the personal network to talk to and be completely honest with. In the study based on UKATT participants’ written statements of what was useful about their sessions, participants stated that having someone to talk to was the most appreciated aspect of treatment, regardless of if they received Social Behavior and Network Therapy or MET [20]. The current results as well as those of the aforementioned study indicate that having a professional to confide in is a highly valued experience shared across treatments, and does not pertain to a certain methodology [19, 20].

In the current study, participants reported that therapists did not interfere with their drinking goals or strategies to approach those goals. Some of the participants described that this approach made them feel like the owner of their problem. This resembles the results of Jones and colleagues, where therapists were perceived to emphasize patients’ autonomy which in turn was important for the patients’ motivation to change [19]. For some of the participants in the current study, treatment resulted in similar experiences. Others described an insufficient level of guidance and desired a higher degree of support on how to approach their goals. One of the aspects that participants who received MET in the UKATT study stated as most helpful, was when they were supported with advice from their therapists. Receiving advice was significantly more often stated as helpful among patients, compared with how often therapists found advising to be helpful to patients. Suppressing the impulse to offer advice is proposed to be part of the MI spirit, but our findings as well as those of the UKATT study, indicate that an overly strict interpretation of this principle may leave some patients feeling unsupported [20]. In cases when patients are not clear on the next step, or have a lack of skills, a menu of options or more specific advice may be used more frequently. To certain patients, a more directive approach may thus be more effective to approach goal setting and planning for change.

Some participants described that there was no room for talking about what they experienced as a failure or negative events, subjects which in their opinion, could have been beneficial to reflect on. A recent study of a brief MI-session addressing risky use of alcohol in emergency units found that discussing negative consequences related to alcohol consumption led to an increase in patients’ perceived readiness to change [29]. It may be hypothesized that a too strong change focus and specifically evocation techniques may be perceived as invalidating to certain patients when delivered with potentially wrong timing. More research on the association between patient expressions of failure and negative events and treatment outcomes in MET, may thus be warranted. It may also be of particular interest to study therapists’ responses to failure and negative events, in order to explore which responses may be the most effective to evoke change talk.

Participants reported that their decision to seek treatment was one step in an ongoing motivational process and that they were already motivated to change at treatment entry. They hoped and expected that treatment would consolidate their wish to change, rather than expecting that the treatment per se would make them change. Seeking treatment may thus have been the most important step in the process from contemplation to readiness for change in these individuals. This is somewhat unexpected, as MI is primarily described as a method which would suit individuals in a pre-contemplation or contemplation stage of change [27]. This is not supported by the current study, where individuals who were committed to change were perceived to be the ones who found treatment most helpful to proceed in their change process.

Participants in this study expressed that the treatment had been helpful regarding both increased awareness and increased control over drinking behavior. However, some had expected to have been more successful in reducing alcohol consumption than they actually were. The general opinion among participants was that they wanted treatment to continue for a longer period than the given 12 weeks to establish and maintain change. A more flexible approach to MET treatment length in clinical settings may thus be beneficial to patients’ sense of self-efficacy.

In the current study, participants were encouraged to bring a SO to the treatment sessions but, interestingly, none of them did. They were, however, in favor of the idea of involving a SO, and some stated that they already had a supporting dialogue with their SO. In practice, most were reluctant to their SO participating in treatment and preferred to interact with their therapist in private. The effect of involving a SO in the treatment of substance use disorders has been examined in relation to several treatment methods [30]. In a meta-analysis [30], the involvement of a SO in treatment was shown to be associated with a reduction of both substance use and problems related to the substance use. In MI for AUD specifically, the involvement of a SO in treatment can lead to more change talk and better treatment outcomes [5, 31]. Despite recommendations of SO involvement in treatment, other studies have also noted that few SOs participate in sessions. In the Project MATCH study, only 15% of patients who received MET brought a SO [32]. The choice not to bring a SO may be linked to the preference to talk to someone outside the family or a fear of unrealistically high expectations and risk of disappointment, as well as being more honest and comfortable without a SO [33, 34]. Similar reasons for not bringing a SO were reported in our study. This implies that it is important to offer an option when it comes to involving SOs in treatment. However, given that SO attendance in treatment has been associated with better treatment outcomes, it may be relevant to inform patients about the benefits of bringing their SO.

## Strengths and limitations

The current study was conducted in an addiction specialist setting, and treatment was performed by highly trained and experienced therapists. By the measures of treatment integrity derived from MITI scores, we could assume that our results were based on MET performed by therapists who were adherent to MITI protocol. Further, this kind of study design gives a good understanding of perceptions in a group of individuals. It does not aim to generalize the results in a quantitative manner. Instead, descriptions of context, process of analysis, and appropriate quotations can inform and enhance readers’ understandings of how the findings can be transferred to other settings or groups.

A limitation to the study is that MET sessions were primarily provided through video meetings instead of face-to-face, due to the COVID pandemic. Some participants expressed a desire to meet their therapist in person, and some also considered the video format to have affected their decision not to bring a SO. However, the video format was not mentioned as the main reason for not bringing a SO. Lastly, one limitation was that participants who were offered to participate in interviews were the ones who retained in the study at the 26-week follow-up. Hence, we did not receive information from participants who dropped out from the study prematurely (16.4%).

## Conclusions

The part of treatment that participants most appreciated was the opportunity to verbalize thoughts and feelings about their problems in a non-judgmental and supportive environment. Notably, they had started their change process before seeking treatment. A less helpful aspect was the non-directive approach to goal-setting, which increased autonomy for some, but was not helpful to others. Furthermore, all participants experienced a need for longer treatment. Lastly, SOs can play an important role as supporters, even though it may not be necessary or desirable to include them in treatment sessions.

### Future directions

Future research on patient experiences from various MI applications in AUD treatments may inform further development of these methods. Research on mechanisms of behavioral change (MOBC) in MI has been suggested to be prematurely narrow, focusing on technical components primarily involving therapist behavior [19, 35]. Qualitative studies on patient perspectives may contribute to this field of further research on MOBC in both MI and specifically in MET, suggesting other mechanisms which may be relevant from the patient’s perspective. Such studies may also generate new MITI-scoring variables to investigate, in relation to treatment outcomes. Moreover, qualitative studies of patient experiences from MET interventions delivered in different formats (e.g., digital, telehealth) may also be of importance, especially as digital formats are becoming more common in healthcare.

## Data Availability

The qualitative data analyzed in the current study is not publicly available. Translated pseudonymized data are available from the corresponding author on request.

## Abbreviations

AUD: Alcohol use disorder
BSCT: Behavioral Self-Control Training
COREQ: Consolidated criteria for Reporting Qualitative research
COVID: Coronavirus disease
DNR: Registry number
FORTE: Swedish Research Council for Health, Working life and Welfare
ISRCTN: International Standard Randomized Controlled Trial Number
MATCH: Matching Alcoholism Treatment to Client Heterogeneity
MET: Motivational Enhancement Therapy
MI: Motivational Interviewing
MITI: Motivational Interviewing Treatment Integrity Code
MOBC: Mechanism of behavior change
RCT: Randomized controlled trial
RSA: Research Council of the Swedish Alcohol Retailing Monopoly
SO: Significant other
UKATT: United Kingdom Alcohol Treatment Trial

## References

[1] Miller WR (2013). Helping people change.

[2] Miller WR (1983). Motivational Interviewing with problem drinkers. Behav Cogn Psychother.

[3] Fried TR, Yang M, Martino S, Iannone L, Zenoni M, Blakley L (2022). Effect of computer-tailored print feedback, motivational interviewing, and motivational enhancement therapy on engagement in advance care planning: a randomized clinical trial. JAMA Intern Med.

[4] Frost H, Campbell P, Maxwell M, O'Carroll RE, Dombrowski SU, Williams B (2018). Effectiveness of motivational interviewing on adult behaviour change in health and social care settings: a systematic review of reviews. PLoS ONE.

[5] Bourke ESM, Magill MPD, Apodaca TRPD (2016). The in-session and long-term role of a significant other in motivational enhancement therapy for alcohol use disorders. J Subst Abuse Treat.

[6] Lundahl B, Burke BL (2009). The effectiveness and applicability of motivational interviewing: a practice-friendly review of four meta-analyses: motivational interviewing and psychotherapy. J Clin Psychol.

[7] Smedslund G, Berg RC, Hammerstrøm KT, Steiro A, Leiknes KA, Dahl HM (2011). Motivational interviewing for substance abuse. Cochrane Database Syst Rev.

[8] Magill M, Apodaca TR, Borsari B, Gaume J, Hoadley A, Gordon REF (2018). A meta-analysis of motivational interviewing process: technical, relational, and conditional process models of change. J Consult Clin Psychol.

[9] Moyers TB, Miller WR, Hendrickson SML (2005). How does motivational interviewing work? Therapist interpersonal skill predicts client involvement within motivational interviewing sessions. J Consult Clin Psychol.

[10] Laws MB, Magill M, Mastroleo NR, Gamarel KE, Howe CJ, Walthers J (2018). A sequential analysis of motivational interviewing technical skills and client responses. J Subst Abuse Treat.

[11] Kramer Schmidt L, Andersen K, Søgaard NA (2022). Differences in the delivery of motivational interviewing across three countries. J Ethn Subst Abuse.

[12] Project Match Research Group (1997). Matching alcoholism treatments to client heterogeneity: project MATCH posttreatment drinking outcomes. J Stud Alcohol.

[13] Team UKATT Research Team (2005). Effectiveness of treatment for alcohol problems: findings of the randomised UK alcohol treatment trial (UKATT). BMJ.

[14] Sellman JD, Sullivan PF, Dore GM, Adamson SJ, MacEwan I (2001). A randomized controlled trial of motivational enhancement therapy (MET) for mild to moderate alcohol dependence. J Stud Alcohol.

[15] Hammarberg A, Andréasson, S, Fahlke, C, Forsberg, L, Johansson, M, Öjehagen A. Manual för motivationshöjande behandling (Motivational Enhancement Therapy). https://alkoholhjalpen.se/sites/default/files/2021-03/Manual_for_Motivationshojande_behandling_MET_1.3.pdf: www.alkoholhjalpen.se; 2015.

[16] Miller WR, Rollnick S (2013). Motivational interviewing: helping people to change.

[17] Hilton CE, Lane C, Johnston LH (2016). Has motivational interviewing fallen into its own premature focus trap?. Int J Adv Couns.

[18] McQueen JM, Ballinger C, Howe TE (2017). Factors associated with alcohol reduction in harmful and hazardous drinkers following alcohol brief intervention in Scotland: a qualitative enquiry. BMC Health Serv Res.

[19] Jones SA, Latchford G, Tober G (2016). Client experiences of motivational interviewing: an interpersonal process recall study. Psychol Psychother.

[20] Orford J, Hodgson R, Copello A, Krishnan M, de Madariaga M, Coulton S (2009). What was useful about that session? Clients’ and therapists’ comments after sessions in the UK alcohol treatment trial (UKATT). Alcohol Alcoholism.

[21] Tong A, Sainsbury P, Craig J (2007). Consolidated criteria for reporting qualitative research (COREQ): a 32-item checklist for interviews and focus groups. Int J Qual Health Care.

[22] Moyers T, Rowell L, Manuel J, Ernst D, Houck J (2016). The Motivational Interviewing Treatment Integrity Code (MITI 4): Rationale, Preliminary Reliability and Validity. J Subst Abuse Treat.

[23] Sobell LC, Maisto SA, Sobell MB, Cooper AM (1979). Reliability of alcohol abusers' self-reports of drinking behavior. Behav Res Ther.

[24] Braun V, Clarke V (2006). Using thematic analysis in psychology. Qual Res Psychol.

[25] Braun V, Clarke V (2021). One size fits all? What counts as quality practice in (reflexive) thematic analysis?. Qual Res Psychol.

[26] Guest G, Bunce A, Johnson L (2006). How many interviews are enough?: an experiment with data saturation and variability. Field Methods.

[27] Miller WR, Rose GS (2009). Toward a theory of motivational interviewing. Am Psychol.

[28] Morgenstern J, Kuerbis A, Amrhein P, Hail L, Lynch K, McKay JR (2012). Motivational interviewing: a pilot test of active ingredients and mechanisms of change. Psychol Addict Behav.

[29] Merrill JE, López G, Stevens AK, Singh S, Laws MB, Mastroleo NR (2022). Discussion of alcohol consequences during a brief motivational intervention session: comparing those who do and do not increase readiness to change. Addi Res Theory.

[30] Ariss T, Fairbairn CE (2020). The effect of significant other involvement in treatment for substance use disorders: a meta-analysis. J Consult Clin Psychol.

[31] Manuel JK, Houck JM, Moyers TB (2012). The impact of significant others in motivational enhancement therapy: findings from project MATCH. Behav Cogn Psychother.

[32] Apodaca TR, Magill M, Longabaugh R, Jackson KM, Monti PM (2013). Effect of a significant other on client change talk in motivational interviewing. J Consult Clin Psychol.

[33] Brobeck E, Odencrants S, Bergh H, Hildingh C (2014). Patients' experiences of lifestyle discussions based on motivational interviewing: a qualitative study. BMC Nurs.

[34] McCrady BS, Epstein EE, Cook S, Jensen NK, Ladd BO (2011). What do women want? Alcohol treatment choices, treatment entry and retention. Psychol Addict Behav.

[35] Longabaugh R (2007). The search for mechanisms of change in behavioral treatments for alcohol use disorders A commentary: the search for mechanisms of behavior change in evidence-based behavioral treatments for alcohol use disorders. Alcoholism Clinical Exp Res.

